# Alterations of Retinal Pigment Epithelium–Photoreceptor Complex in Patients with Type 2 Diabetes Mellitus without Diabetic Retinopathy: A Cross-Sectional Study

**DOI:** 10.1155/2020/9232157

**Published:** 2020-03-06

**Authors:** Zheren Xia, Hao Chen, Suilian Zheng

**Affiliations:** Department of Ophthalmology, The Second Affiliated Hospital and Yuying Children's Hospital of Wenzhou Medical University, Wenzhou, Zhejiang, China

## Abstract

**Aim:**

A cross-sectional study was performed to examine the alterations of the retinal pigment epithelium– (RPE–) photoreceptor complex layer in type 2 diabetes mellitus (DM) without diabetic retinopathy (DR), using spectral-domain optical coherence tomography (SD-OCT).

**Methods:**

Patients with type 2 DM without DR and healthy controls without DM were recruited. All participants underwent examinations including SD-OCT. The thickness measurements of the retinal neural layers were calculated after automatic segmentation. An independent-sample *t*-test was used to compare the means of the thickness of retinal neural layers in patients with DM and healthy controls.

**Results:**

Sixty-seven eyes from 67 patients with DM and 30 eyes from 30 healthy controls were included in this study. No significant differences were found in age (*P* = 0.601), gender (*P* = 0.601), gender (*P* = 0.601), gender (*P* = 0.601), gender (*P* = 0.601), gender (*P* = 0.601), gender (*P* = 0.601), gender (*P* = 0.601), gender (

**Conclusion:**

Lesions in the RPE–photoreceptor complex are present without vascular abnormalities, which may precede the alterations of ganglion cells in patients with type 2 DM.

## 1. Introduction

Diabetic retinopathy (DR) caused by type 2 diabetes mellitus (DM) is one of the major complications leading to blindness. Microvascular abnormalities have received close attention, whereas neurodegeneration has recently been reported to precede visible vascular lesions [1–3]. The development of spectral-domain optical coherence tomography (SD-OCT) has allowed imaging and measuring of the retinal layers with high accuracy after automated three-dimensional segmentation [4, 5]. With built-in software, each retinal layer is able to be identified automatically. Thus, investigators began to explore the changes in OCT images to verify the presence of early neural dysfunction. They discovered the damages of the retinal nerve fiber layer (RNFL) and ganglion cell–inner plexiform layer or ganglion cell layer (GCL) [6–8]. Some other studies [9–12] have focused on the outer layers of the retina, such as the outer nuclear layer (ONL), inner segment photoreceptors, or outer segment photoreceptors (OS). However, these studies have noted inconsistent results.

Neural alterations are considered to be the early signs of retinal dysfunction in patients with type 2 DM. Therefore, this study aimed to determine whether or not there are alterations in the retinal neural layers, including the retinal pigment epithelium (RPE)–photoreceptor complex or ganglion cells of patients with type 2 DM without any clinical DR, using SD-OCT.

## 2. Materials and Methods

### 2.1. Participants

This cross-sectional study was performed between July 2016 and February 2018. Sixty-seven eyes from 67 patients with type 2 DM without DR were included in the DM group, and 30 eyes from 30 healthy controls were recruited in the control group. Unless the image quality of the left eye was better, the right eye was considered for analysis. Patients were recruited from the outpatient service and inpatient ward of the Department of Ophthalmology at the Second Affiliated Hospital and Yuying Children's Hospital of Wenzhou Medical University (Wenzhou, China).

Inclusion criteria were patients with type 2 DM without DR evaluated by a retinal specialist through an ophthalmoscope and color fundus photographs (TRC-50DX; Topcon, Tokyo, Japan) after pupil dilation. All participants underwent a comprehensive examination, including the best-corrected visual acuity (BCVA), intraocular pressure (IOP) measured by a noncontact tonometer (TX-20P; Canon, Tokyo, Japan), axial length measured by the IOL Master (Carl Zeiss AG, Oberkochen, Germany), SD-OCT examination (Heidelberg Engineering, Heidelberg, Germany), and hemoglobin A_1c_ (HbA_1c_) value examined from a blood sample obtained at the time of ophthalmic examinations. The patient information including the duration of diabetes and date of birth was dictated by patients. Exclusion criteria were as follows: (a) myopia greater than −6D or hyperopia greater than +3D; (b) BCVA less than 0.1; (c) significant media opacity that causes the inaccuracy or failure of automatic segmentation; (d) history or presence of glaucoma, uveitis, pathological myopia, optic neuropathy, retinal detachment, and any other nondiabetic retinopathy; (e) presence of macular diseases; and (f) history of retinal surgeries.

Normal controls were healthy patients from the outpatient service without any ocular or systemic diseases. This study obeyed the tenets of the Declaration of Helsinki. All participants were given an informed consent form.

The study was approved by the Research Ethics Committee of the Second Affiliated Hospital of Wenzhou Medical University.

### 2.2. Optical Coherence Tomography Imaging and Layer Segmentation

**Figure 1:**
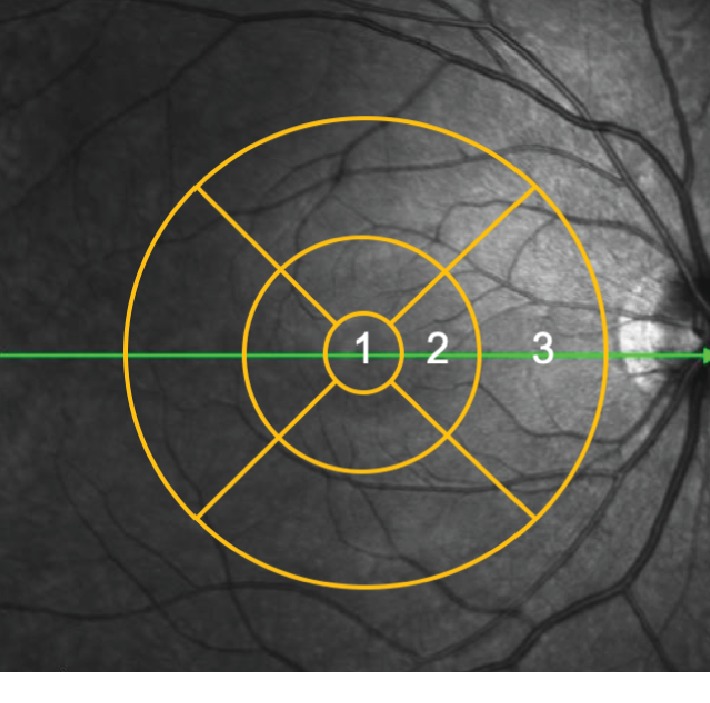
Three concentric circles of 1, 3, and 6 mm diameter with the center of the macular fovea and nine Early Treatment Diabetic Retinopathy Study areas. 1, foveal area; 2, paracentral area; and 3, peripheral area.

**Figure 2:**
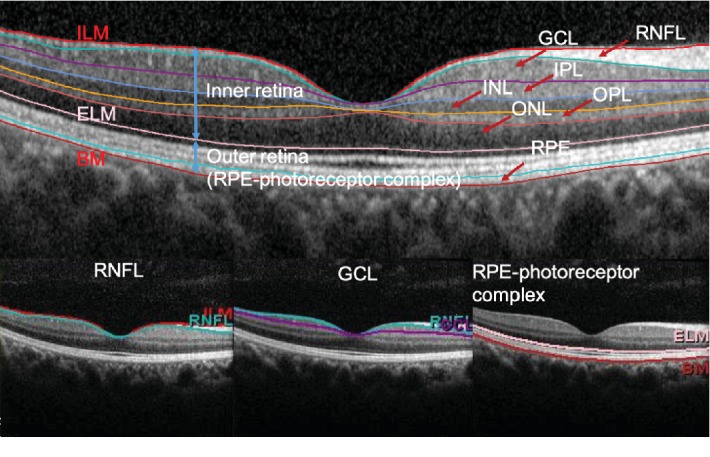
The retina was automatically segmented into nine layers: retinal nerve fiber layer (RNFL), ganglion cell layer (GCL), inner plexiform layer (IPL), inner nuclear layer (INL), outer plexiform layer (OPL), outer nuclear layer (ONL), retinal pigment epithelium (RPE), inner retinal layers, and outer retinal layers (RPE–photoreceptor complex).

SD-OCT examination was performed to obtain 20° × 20° macular cube scans (49 B-scan sections, 120 *μ*m spacing, and 512 A/B scans), which were divided into three concentric circles of 1, 3, and 6 mm diameter with the center of the macular fovea. These three circles were further subdivided into nine Early Treatment Diabetic Retinopathy Study areas (Figure 1). The retina was automatically segmented, and the thicknesses of nine layers were measured by built-in software: RNFL, GCL, inner plexiform layer (IPL), inner nuclear layer, outer plexiform layer, ONL, RPE, inner retinal layers (from inner limiting membrane to external limiting membrane), and outer retinal layers (from external limiting membrane to Bruch's membrane), which we defined as the RPE–photoreceptor complex layer (Figure 2). The sectoral (superior, inferior, temporal, and nasal) thickness of each layer in the paracentral area (with an inner diameter of 1 mm and outer diameter of 3 mm) and thickness of the foveal area (with an outer diameter of 1 mm) were calculated automatically. In this study, we mainly discussed the mean thicknesses of RNFL, GCL, and RPE–photoreceptor complex in the foveal and paracentral areas.

According to the manufacturer guidelines, only images that had higher than 25 dB (ranges from 0 to 40) of quality score were included. The OCT images were reviewed by an experienced retinal specialist, and segment error (such as the lines were not corresponding to the proper retinal layers) or low quality, if any, was excluded.

### 2.3. Statistical Analysis

An independent-sample *t*-test was performed to evaluate differences in age, axial length, IOP, and thicknesses of the RPE–photoreceptor complex layer, RNFL, and GCL between the DM and control groups. Gender difference between the two groups was compared using a chi-squared test. The Mann–Whitney *U* test was used to compare differences in BCVA and HbA_1c_ between the two groups.

The repeatability of the measurements was accessed for both DM patients and controls. Ten eyes of both groups were enrolled. The measurements were performed twice by a single operator at different times. The intraclass correlation coefficients (ICCs) were calculated to evaluate the repeatability.

All statistical tests were two-sided. A *P* value of <0.05 was considered statistically significant, and statistical analysis was performed using SPSS (v23.0; IBM Corp., Armonk, NY).

## 3. Results

Sixty-seven eyes from 67 patients with DM were included in the DM group. Of these 67 patients, 34 were male and 33 female patients. The mean age was 57.20 ± 13.84 years. The mean axial length of the eyes was 23.30 ± 0.64 mm. The mean duration of DM was 4.0 years (ranging from 2.0 to 9.0 years). The mean HbA_1c_ value was 7.2% (ranging from 6.6 to 9.5%). The mean BCVA was 1.0 (ranging from 0.7 to 1.0). The mean IOP was 14.93 ± 3.41 mmHg.

Thirty eyes from 30 healthy controls were included. Of these 30 controls, 15 were male and 15 female, with a mean age of 58.70 ± 10.62 years. The mean axial length of the eyes was 23.44 ± 0.83 mm. The mean HbA_1c_ value was 5.6 ± 0.2%. The mean BCVA was 0.9 (ranging from 0.8 to 1.0). The mean IOP was 13.70 ± 3.70 mmHg. No significant differences in age (*P* = 0.601), gender (*P* = 0.560), axial length (*P* = 0.414), BCVA (*P* = 0.963), or IOP (*P* = 0.112) were found between the DM and control groups. The HbA_1c_ value of the DM group was higher than that of the control group (*P* < 0.001; Table 1).

The ICCs obtained of the two groups were as follows: RNFL: 0.99, GCL: 0.99, RPE–photoreceptor complex: 0.96 (DM group); RNFL: 0.99, GCL: 0.99, RPE–photoreceptor complex: 0.94 (control group), demonstrating good repeatability of the measurements.

There were statistically significant increases in the thickness of the RPE–photoreceptor complex in the foveal area (*P* = 0.027) and paracentral area (*P* = 0.001) of patients with DM compared to healthy controls. However, patients with DM showed slight, but not statistically significant, decreases in the mean thickness of RNFL in the foveal area (*P* = 0.713) and paracentral area (*P* = 0.319) and GCL in the foveal area (*P* = 0.454) and paracentral area (*P* = 0.472) when compared to healthy controls.

The mean thicknesses of the RPE–photoreceptor complex, RNFL, and GCL in the foveal and paracentral areas of patients with DM and healthy controls are provided in Table 2.

## 4. Discussion

In this study, we reported an increase in the thickness of the RPE–photoreceptor complex in the macula of diabetic eyes without any sign of DR. However, no changes in the thickness of RNFL and GCL were detected.

RPE is a monolayer of pigmented cells playing an essential role in photoreceptor function, survival, and maintenance [13]. Light-sensitive outer segments of photoreceptors surrounded by long apical microvilli of RPE cells form a complex of tight interaction [14]. These multiple close interactions are associated with many important functions of the outer retina, including the recovery of photoreceptor sensitivity after a bleach [14]. Thus, RPE and photoreceptor can be regarded as a functional unit because both tissues depend on each other. The structural and functional changes of the RPE–photoreceptor complex have been demonstrated in diabetic macular edema previously [15]. However, to the best of our knowledge, few studies have reported the alterations of the RPE–photoreceptor complex in patients with DM without any clinically detectable DR.

Nesper et al. [16] have illuminated that choriocapillary nonperfusion occurs more frequently in the eyes of patients with DM than those of healthy controls, using OCT angiography (OCTA). Muir et al. [17] have demonstrated that the reduction of choroidal blood flow could be an early pathological change in DR. These pieces of evidence indicate that photoreceptors and RPE cells are exposed to a relatively hypoxic environment, as choroidal vessels are essential in supplying oxygen, water, ions, and nutrients to RPE and photoreceptors [18]. Hypoxia has been reported to induce the dysfunction of phagocytosis and fragility of RPE cells [19]. Additionally, photoreceptors—the most metabolically active neurons in the central nervous system [20]—would produce increased superoxide and soluble inflammatory factors in elevated glucose [21, 22]. These factors may cause a malfunction of RPE as well.

RPE cells digest the shed OS, returning essential substances to photoreceptors. Phagocytosis always occurs on the apical membranes of the RPE cells facing photoreceptor cells [23]. Because of a high number of photoreceptors per RPE cell in the macula, RPE cells in this region are adjusted to a high turnover rate of the renewal of OS [24]. Thus, we hypothesized that the disturbance of phagocytosis of RPE cells would induce the accumulation of shed OS that is not timely engulfed in the RPE–photoreceptor complex. This could be a reasonable explanation for the increase in the RPE–photoreceptor complex layer in the macular area.

Alterations of the RPE–photoreceptor complex were consistent with evidence from visual electrophysiology and color vision. Schneck et al. [25] have demonstrated that the fast oscillation of the electrooculogram (EOG) was reduced in retinopathy-free patients with type 2 DM, which depended on the integrity of photoreceptors and RPE. Likewise, color vision was recognized to be impaired in patients with no clinical DR [26–29].

Some studies have focused on particularly the outer retina layers in diabetic retinopathy. Gella et al. [9] and Verma et al. [10] have found that photoreceptor (PR) layer thickness significantly decreased in diabetic subjects without DR compared to healthy controls, with the Copernicus OCT and a manual measurement method. However, Wanek et al. [11] did not find significant difference in OS and RPE between subjects without DR and controls with the Spectralis OCT and a customized image segmentation method. Ferreira et al. [12] have reported a thicker RPE and a thinner PR in diabetic patients without DR when compared with nondiabetic controls with the built-in automatic segmentation software of the Spectralis OCT and proposed that the retinal thickness did not have linear relationship with the duration of diabetes (patients with longest diabetes duration had thicker PR thickness than those of moderate duration). In the current study, the RPE–photoreceptor complex thickness was measured as a whole and was significantly increased in diabetic subjects without DR. The types of OCT machine, the segmentation algorithms of the outer retina, the DM duration, and the divisions of retinal area would display different results. A multicenter and standardized study is anticipated in the future.

In this study, unlike previous studies [6–8], we did not find any alternation in RNFL or GCL, using SD-OCT. Thus, we assumed that lesions in the RPE–photoreceptor complex preceded the loss of ganglion cells in the diabetic retina without microvascular abnormalities, as the duration of diabetes in this study was shorter than that in previous studies. Further investigations are required to confirm this finding.

Limitations of the this study should be discussed: (a) the relatively small sample size of the DM group could have affected potential associations; (2) the thickness of the RPE–photoreceptor complex, RNFL, and GCL in the peripheral area (with an inner diameter of 3 mm and outer diameter of 6 mm) was not analyzed in this study; and (3) OCTA evaluating choroid blood flow and EOG and color vision test evaluating the function of the RPE–photoreceptor complex were not tested in our patients with DM. Besides, fundus fluorescein angiography was not performed.

## 5. Conclusions

The increase of the thickness of the RPE–photoreceptor complex layer in the macular area occurred, whereas no vascular lesion was detected. Furthermore, no changes in the thickness of RNFL and GCL were detected in this study, indicating that lesions in the RPE–photoreceptor complex may precede the alterations of RNFL or GCL. This assumption needs further studies to confirm. In future clinical work, besides the thickness of RNFL and GCL, the thickness of the RPE–photoreceptor complex could be an important assessment of the retinal neural damage of patients with DM.

## Figures and Tables

**Table 1:** Comparison of characteristics between the DM and control groups.

	Patients with DM	Healthy controls	*P* value
Number of eyes (patients)	67 (67)	30 (30)	—
Age (years)	57.20 ± 13.84^a^	58.70 ± 10.62^a^	0.601
Male : female	34 : 33	15 : 15	0.560
Duration of DM (years)	4.0 (2.0–9.0)^b^	—	—
HbA_1c_ (%)	7.2 (6.6–9.5)^b^	5.6 ± 0.2^a^	<0.001
Axial length (mm)	23.30 ± 0.64^a^	23.44 ± 0.83^a^	0.414
BCVA	1.0 (0.7–1.0)^b^	0.9 (0.8–1.0)^b^	0.963
IOP (mmHg)	14.93 ± 13.84^a^	13.70 ± 3.70^a^	0.112

Abbreviations: DM: diabetes mellitus; HbA_1c_: hemoglobin A_1c_; BCVA: best-corrected visual acuity; IOP: intraocular pressure. ^a^Mean ± standard deviation. ^b^Median (interquartile range).

**Table 2:** Mean thickness measurements (*μ*m) of the RPE–photoreceptor complex, RNFL, and GCL in the foveal and paracentral areas of patients with DM and healthy controls.

Layers	Thickness (mean)		95% CI of the difference	*P* value
	Patients with DM (*μ*m)	Healthy controls (*μ*m)		
RNFL_F	12.31 ± 2.28	12.50 ± 2.35	−1.19, 0.82	0.713
RNFL_PC	22.75 ± 2.65	23.30 ± 2.18	−1.65, 0.54	0.319
GCL_F	14.70 ± 4.21	15.60 ± 7.51	−3.27, 1.47	0.454
GCL_PC	49.87 ± 5.64	50.69 ± 4.06	−3.10, 1.44	0.472
RPC_F	89.37 ± 4.20	87.43 ± 3.19	0.23, 3.65	0.027^∗^
RPC_PC	81.46 ± 2.52	79.61 ± 2.23	0.79, 2.91	0.001^∗^

Abbreviations: F: foveal area; PC: paracentral area; RPC: RPE–photoreceptor complex; CI: confidence interval; RNFL: retinal nerve fiber layer; GCL: ganglion cell layer; RPE: retinal pigment epithelium. ^∗^*P* < 0.05 shows a significant difference.

## Data Availability

The data used to support the findings of this study are available from the corresponding author upon request.
